# Hidradenitis Suppurativa and Reddit: Assessing Gaps in Patient Education

**DOI:** 10.7759/cureus.83736

**Published:** 2025-05-08

**Authors:** Vineeth R Vaidyula, Samantha Ouellette, Cooper Lathrop, Cindy Wassef

**Affiliations:** 1 School of Medicine, Icahn School of Medicine at Mount Sinai, New York, USA; 2 Department of Dermatology, Rutgers Robert Wood Johnson Medical School, Piscataway, USA

**Keywords:** autoimmune disease management, hidradenitis suppurativa, patient education, reddit, social media

## Abstract

Background: Hidradenitis suppurativa (HS) is a chronic autoinflammatory skin condition distinguished by recurrent lesions, including nodules, sinus tracts, and abscesses, in intertriginous sites. HS can lead to low self-esteem and social isolation and may have deleterious effects on a patient's quality of life. HS continues to be insufficiently controlled leading patients to utilize health-related social media and online forums for educational, emotional, and social support. The open-access forum, Reddit, has become a popular outlet for patients and is a valuable source for identifying potential gaps in patient education.

Materials and methods: Information from posts on the “Hidradenitis” Reddit page from October 14, 2021, to January 30, 2022, was extracted using the web scraping tool, ParseHub. Post content was analyzed, and questions were sorted into the following categories: “uncertainty about symptoms,” “medications,” “procedures,” “non-pharmacological management,” “non-specific management,” “causes and triggers,” and “miscellaneous.”

Results: A total of 650 questions from 617 posts were analyzed. Posts had an average of 7.1 upvotes and 9.2 comments. The number of questions per category was as follows: nonpharmacological management (139 (21.4%)), non-specific management (127 (19.5%)), miscellaneous (125 (19.2%)), medications (90 (13.9%)), uncertainty about symptoms (69 (10.6%)), causes and triggers (57 (8.8%)), and procedures (43 (6.6%)).

Conclusion: Our analysis of patient questions posted to the HS subreddit revealed that most patient questions were related to disease management, with non-pharmacological management being the most asked-about category. Providers can address this potential gap in patient education by inquiring about helpful products and practices from their patients and serving as a conduit for specific recommendations that align with the literature.

## Introduction

Hidradenitis suppurativa (HS) is a chronic autoinflammatory skin condition distinguished by recurrent lesions in intertriginous sites. Lesions can include deep-seated nodules, draining sinus tracts, and abscesses, and are often malodorous, painful, and suppurative [1]. Compared to patients without HS, patients with HS are two times more likely to experience anxiety, are 2.5 times more likely to experience depression, and have a greater prevalence of suicide overall [2].

The treatment of HS is challenging due to the chronic and relapse-remitting nature of HS. The high incidence of emergency department admissions and inpatient hospitalizations of HS patients demonstrate that HS continues to be insufficiently controlled, leaving patients discouraged and dissatisfied with their disease management [3]. As a result, patients often turn to health-related social media and online forums for additional educational, emotional, and social support [4]. Reddit is a popular social media outlet where patients can discuss, share, and receive health-related information anonymously. The platform is organized into “subreddits”, which are open-access forums devoted to discussing specific topics. The r/Hidradenitis subreddit is an actively growing community that boasts 40,485 subscribers as of August 2024, up from 2,741 in December 2018 [5].

As the popularity of Reddit among patients continues to grow, new research is emerging that utilizes Reddit as a source for first-hand accounts of patient experiences. Previous studies have performed content analyses on user posts regarding atopic dermatitis, acne, and psoriasis; however, an analysis of HS posts has not been performed [6,7]. The objective of this study is to understand the information needs of HS patients via a content analysis of questions posted to Reddit.

## Materials and methods

This study was a qualitative content analysis aimed at identifying and categorizing key patient questions related to HS. The Rutgers University Institutional Review Board (IRB) deemed this study exempt from IRB review as the data used in this study was collected from a publicly accessible forum.

Publicly accessible posts uploaded to the r/Hidradenitis subreddit between October 14, 2021, and January 30, 2022, were collected using the web scraping tool, ParseHub. Username, title, text, URL, upvote number (approval rating), comment count, and date were extracted from each post. Posts that did not contain questions or HS-related content were excluded from the content analysis. Additionally, posts written in languages other than English and posts asking for users to aid in new diagnoses of HS were excluded. Questions within each post’s title and text were manually reviewed and recorded. Reviewers then categorized each question into one of seven themes similar to those published in previous Reddit analyses: “non-pharmacological management,” “non-specific management,” “miscellaneous,” “medications,” “uncertainty about symptoms,” “causes and triggers,” and “procedures” [6,8]. The miscellaneous category was reserved for any additional themes identified during analysis that contained less than 5% of the total questions.

Data collected using Parsehub were compiled into a Microsoft Excel (Microsoft Corp., Redmond, WA) spreadsheet. The dataset included only publicly available information and excluded any personally identifiable details beyond Reddit usernames. All files were stored on a secure, password-protected computer accessible only to the research team. To ensure anonymity, usernames were not linked to any external identifiers, and all analysis was conducted using de-identified data.

## Results

A total of 650 questions from 617 posts were analyzed, as some posts contained multiple questions. The number of posts by category is summarized in Table 1 and example posts by category are listed in Table 2. Posts had an average of 7.1 upvotes and 9.2 comments.

**Table 1:** Number of questions per category.

Category	Number of questions (% of total questions)
Non-pharmacological management	139 (21.4)
Non-specific management	127 (19.5)
Miscellaneous	125 (19.2)
Medications	90 (13.9)
Uncertainty about symptoms	69 (10.6)
Causes and triggers	57 (8.8)
Procedures	43 (6.6)

**Table 2:** Example posts by category. AHA/BHA: alpha hydroxy acid/beta hydroxy acid; KP: keratosis pilaris; HS: hidradenitis suppurativa.

Category	Subcategory	Post question
Non-pharmacological management	Personal hygiene	What does everyone do to remove their hair from the area they suffer with HS?
	Clothing	Does anyone know any good bras that don’t bother armpits so much?
	Over-the-counter skincare	AHA/BHA serums and creams have helped drastically with my KP and acne. Wondering if maybe they could help with the HS too?
	Diet	For those of you who have had success reducing your symptoms or putting your HS into remission by following a ketogenic diet, how long did it take for you to see improvements?
	Supplements	Should I add supplemental iron or other vitamins and minerals to my diet to offset this low-level continuous loss (of blood)?
Non-specific management	General management	Just wanted to ask if any of you Lovely people know of ways to prevent flare-ups… and how do you best take care of your skin to prevent this from getting worse?
	Lesion-specific care	My HS is… on my labia. I use clobetasol and that isn't doing anything. Aside from contacting my derm tomorrow, does anyone have any relief methods?
	Exercise and hygiene	I've been reading a lot about sweat and flares on here, but does anyone have a care routine for working out?
Miscellaneous	Seeking social support	Sometimes I have thoughts wishing I had a different disease, even really bad ones. Anyone relate?
	Social perception	Have you found guys or girls that don't find this repulsive?
	Financial and employment issues	I was wondering what do you tell your employers on days you call out sick due to a boil?
	Mental health	We often talk about how to heal and treat the disease but how do you self-care to distract you from it being all you think about?
	Doctor-patient relationship	Because of some past trauma I have a really hard time with the whole being totally naked in the doctor's office thing…I’m wondering - does anyone have any advice on how to handle…not wanting certain areas of my body to be looked at or touched?
	Comorbidities	Just wondering if anyone else that has HS also struggles with psoriasis. Is there any connection between the two?
	Provider recommendations	Anyone have any resources on finding a dermatologist well-versed in HS?
Medications	Hormonal	Will the birth control help my HS?
	Steroids	Wondering if anyone has used [triamcinolone cream]? Wanted to know if it worked well or just clogged your pores. Thinking about applying it to a cyst.
	Biologics	Has anyone else here tried Humera and what have your experiences been like?
	Antibiotics	Do you guys think amoxicillin will help [my flare]? Almost every other antibiotic I try (doxycycline, clindamycin, etc) notoriously doesn’t do anything for me.
	Retinoids	Does Accutane/isotretinoin cure/help with HS?
	Pain medications	Any suggestions or remedies for pain? I can't take Advil or anything in that drug class and Tylenol does not work.
	Antibacterials/ antiseptics	Can you use 4% chlorhexidine on active flare-up? If so what is the best way to use it?
Uncertainty about symptoms		Is there anything on {HS} causing dry lips?
Causes and triggers	How to find triggers	Any tips to find triggers and avoid getting flair-ups?
	Examples of causes and triggers	Just wondering if any of you experience HS when gaining weight and/or smoking?
Procedures	Surgeries	Should I try doxycycline or try getting an excision? What is the healing time and pain like?
	Lasers	Will laser surgery even help with (groin HS)?

Non-pharmacological management

The most common category of questions was non-pharmacological management with 139 (21.4%) questions. Questions in this category mainly related to personal hygiene, clothing and accessories, over-the-counter (OTC) skincare, diet, and non-medicative supplements. Among personal hygiene questions, individuals asked questions about HS-specific rituals, including shaving/hair removal, band-aid application, and bathing practices. Among clothing questions, individuals sought recommendations for types of clothing to wear on top of flared skin, including clothing that was leakproof and reduced friction. For OTC skincare, individuals asked questions about substances often marketed for HS and erythematous skin, including turmeric, tea tree oil, emuaid, camphor with eucalyptus oil and menthol, zinc oxide, epsom salts, vaseline, cannabis, and apple cider vinegar. Questions about wound dressings included those about medihoney ointment, silver ointment, hydroseal bandaids, boil oil, amerigel hydrogel, clay masks, and latex-free bandaids. Approximately half of the questions pertaining to diet asked about types of diet plans to adopt to manage HS. Diet plans included the autoimmune protocol (AIP) diet, ketogenic diet, Wahls diet, carnivore diet, intermittent fasting, and caloric-restricted fasting. The other half of the diet questions were regarding advice about whether certain foods were related to HS onset and flares.

Non-specific management

The second most common category of questions was non-specific management, with 127 (19.5%) questions. In this category, patients inquired about general advice for managing HS symptoms. Most individuals focused on the general management of flares and disease progression, but individuals also sought advice for managing pain, draining, cleaning, and refilling boils, and managing surfacing and popped lesions. Additionally, several individuals requested advice on how to manage hygiene while exercising. The most asked-about locations of HS lesions included the forehead and groin areas.

Miscellaneous

The miscellaneous category was comprised of 125 (19.2%) questions. Questions in this category were focused mainly on seeking social support, social perception, financial and employment issues, mental health, doctor-patient relationships, comorbidities, and provider recommendations. In the seeking social support and social perception categories, individuals were concerned about resuming normal, daily activities due to embarrassment. Individuals requested advice for telling loved ones about their HS and finding resources and support circles. Similarly, individuals who asked questions in the financial employment issues category sought out advice for working while living with HS. In the mental health category, individuals asked about others’ experiences of stress, depression, and suicidal ideation and ways to cope and practice self-care. The doctor-patient relationship category entailed individuals asking about their physician potentially misdiagnosing their HS and about uncomfortableness while undressing in the clinic. In the laboratory tests category, individuals sought out advice for elevated counts on blood tests and food sensitivity tests. Individuals requested advice about possible HS comorbidities, including Crohn’s disease, cardiovascular complications, and polycystic ovarian syndrome. Several individuals were also concerned about HS heritability, HS impact on immunity, and disease progression and remission later in life. Additionally, individuals sought advice about what type of doctor to see for their HS, including dermatologists, plastic surgeons, general practitioners, and alternative medicine providers.

Medications

Ninety (13.9%) questions were classified into the medications category. This category was divided into the following subcategories: hormonal, steroids, biologics, antibiotics, retinoids, pain medications, and antibacterials/antiseptics. In the biologics subcategory, the majority of individuals asked about adalimumab, while others inquired about guselkumab, infliximab, and ustekinumab. Individuals requesting advice about biologics were mainly concerned with drug efficacy, tolerability, and side effects. In the hormonal subcategory, individuals sought advice about oral contraceptives and other hormonal birth control medications. Most individuals were concerned about using hormonal contraceptives to manage flare-up severity, while others inquired about the safety of using hormonal contraceptives with HS. Additionally, patients asked about antiandrogen medications, such as clascoterone. In the antibiotics subcategory, the most frequently asked about medication was doxycycline, followed by clindamycin, rifampin, amoxicillin, lymecycline, metronidazole, moxifloxacin, and dapsone. Some individuals were concerned about antibiotic resistance from continuous use. Individuals seeking advice in the antibacterial/antiseptic category inquired about chlorhexidine gluconate and dilute sodium hypochlorite. Individuals were interested in people’s experiences with the antiseptics on lesions, as well as chlorhexidine alternatives due to irritation. Additionally, individuals requested advice about isotretinoin in the retinoids category and cortisone and general analgesics in the pain medications category. Other medications that were inquired about included spironolactone, immunosuppressants, metformin, botulinum toxin, iron and probiotic supplements, benzoyl peroxide, and hydroquinone for hyperpigmentation. Across all medication subcategories, some individuals sought advice about the safety and efficacy of using two or more medications at the same time.

Uncertainty about symptoms

Sixty-nine (10.6%) questions were categorized as uncertainty about symptoms. Most individuals asking questions in this category were concerned about whether a symptom they were experiencing was characteristic of HS. The majority of questions were related to lesions. In descending order by the number of questions asked, the remaining symptoms inquired about were flares, bleeding, nausea, pain, itchiness, swelling, and cellulitis.

Causes and triggers

Fifty-seven (8.8%) questions were related to the causes and triggers of HS. Individuals sought advice on how to find their triggers, while others questioned if the cause of their HS symptoms was related to diet components, weight, hormones (including questions regarding menstruation, pregnancy, estrogen supplements, general hormones, and c-section birth), weight, and smoking.

Procedures

Forty-three (6.6%) questions made up the procedures category. This category was divided into two subcategories: surgeries and lasers. In the surgeries subcategory, individuals asked questions about general surgery experiences and specific surgeries including excision, deroofing, marsupialization, and incision-and-drainage. Individuals were most interested in post-procedure care and symptoms, including bruising, bleeding, and pain. Similar questions about others’ experiences with laser therapies, including general, hair removal, and CO_2_ lasers, were asked in the laser subcategory. Hair removal was a popular topic as well, with individuals asking about laser, electrolysis, and general removal. Other procedures that were asked about included scar removal, light-emitting diode (LED) light therapy, and pain prevention procedures.

## Discussion

This content analysis of Reddit has revealed valuable information related to HS patient concerns, educational gaps, and disease management. Non-pharmacological management was the most prevalent category demonstrating that patients most sought clothing, wound dressing, personal hygiene, skincare, diet, and supplement recommendations. Non-specific management was the second most common category, and this category was comprised of questions about how to manage HS without mention of specific treatments. Together, these two top categories, along with the medications category, represent approximately half of the total questions asked. While dermatologists often focus primarily on pharmacological and surgical management, these findings highlight that patients desire more information on day-to-day activities such as hygiene, wound care, and clothing, among other things. Providing more information regarding specific recommendations for this topic is one initiative that can help address patient questions. Further studies would be helpful to discuss the knowledge base dermatologists have regarding these non-medical treatments as a knowledge gap may exist.

A systematic review in 2021 focused on studies related to various non-pharmacological recommendations for HS [9]. For clothing, non-abrasive clothing is recommended to reduce friction, as friction can promote new lesion formation [10-12]. Additionally, loose, breathable fabric, such as cotton, is preferred to maintain a cooler and dryer environment that discourages bacterial growth [12-15]. Dressing recommendations depend on various factors but should adequately absorb fluids, with options ranging from superabsorbent foams and hydrofibers to calcium alginates depending on the amount of exudate [16]. Importantly, dressings should also minimize friction and utilize adhesives that cause less pain during dressing changes [16]. Although the literature provides recommendations for the type of fabric to wear or dressings to buy, patients may benefit from recommendations for specific products and brands. As many patients end up turning to the Reddit community for these recommendations, providers can help streamline this information by asking HS patients about success with specific non-pharmacological products and ensuring they align with the literature. Regarding hygiene recommendations, HS is exacerbated by heat and sweat, therefore patients should rinse with water after exercise or prolonged exposure to warm, humid environments [13,14]. While warm baths alone have been reported to help alleviate HS symptoms, the addition of ¼ cup of hypochlorous acid has been shown to reduce bacterial load and odor, as well as pain and itch. The link between shaving and HS is not well understood; however, some patients have reported shaving as an exacerbating factor [13,17].

While the link between obesity and HS has been studied extensively, there is a dearth of studies on specific dietary interventions. The influence of zinc, vitamin D, and vitamin B12 supplementation on HS has been previously reported. In a systematic review of dietary interventions for HS conducted in 2020, studies showed significant improvement in HS lesions with myo-inositol 2000 mg, folic acid, and liposomal magnesium once daily, zinc gluconate 90 mg once daily, and vitamin D supplementation in HS patients with vitamin D deficiency [18-22]. This is consistent with data showing that the severity of vitamin D deficiency and lower zinc levels correlate to more severe disease as assessed by the Hurley stage [22]. Two smaller case series, one studying 1,000 μg vitamin B12 intramuscular every two weeks and the other studying 3 mg riboflavin (vitamin B2) three times daily, also reported promising results [23,24]. Regarding overall diet, one study highlighted lower complex carbohydrate and higher saturated fatty acid intake observed in HS subjects compared to controls [25]. Moreover, adherence to a Mediterranean diet has been shown to correlate with lower HS severity [25]. Additionally, studies demonstrated that the exclusion of dairy, wheat, and brewer’s yeast may also be beneficial to some HS patients [26].

Regarding specific therapies for HS, there is a lack of high‐quality evidence to guide treatment decisions. The potential role of hormones in HS is not yet well understood, but an imbalance of progesterone, estradiol, and/or androgens are all likely implicated in HS pathogenesis. There have been very few studies conducted assessing hormonal contraceptives as a therapy for HS and the results are conflicting. The largest study to date was a cohort study of 100 women of childbearing age in Granada, Spain, taking either progestin-only or combined oral contraceptive pills (OCPs) for HS who were found to have a significant reduction in abscess and nodule count compared to patients not taking OCPs [27]. This study also found that shorter HS disease duration, perimenstrual flares, and concomitant therapy with oral doxycycline or topical clindamycin were independently associated with increased OCP effectiveness. In regard to other antiandrogen therapy for HS, while positive results have been reported with finasteride, dutasteride, and spironolactone, a metanalysis in 2019 found that current studies do not allow a robust evidence-based recommendation for the use of antiandrogens for HS and highlighted the need for further randomized controlled trials [28].

Doxycycline is an antibiotic prescribed for the management of HS, however high-quality studies are lacking. In a prospective study of 108 HS patients in Denmark treated with tetracycline (500 mg twice daily), doxycycline (100 mg twice daily), and lymecycline (300 mg twice daily), there was a significant decrease in mean Hidradenitis Suppurativa Score (HSS), with no significant differences between the three groups [29]. Additionally, a significant decrease in the mean Dermatology Life Quality Index (DLQI) score and overall disease-related distress Numerical Rating Scale (NRS) was observed in all patients. In a separate international cohort study of 283 patients either receiving a tetracycline antibiotic (tetracycline, doxycycline, or minocycline) or clindamycin plus rifampin, both groups showed a significant decrease in the International Hidradenitis Suppurativa Scoring System (IHS4) [30]. There was no significant difference in the percentage of patients achieving Hidradenitis Suppurativa Clinical Response (HiSCR) (defined as >50% reduction in inflammatory lesion count and no increase in abscesses or draining fistulas compared with baseline). In both studies, the most reported side effects for tetracycline antibiotics were gastrointestinal.

## Conclusions

HS is a chronic autoinflammatory skin characterized by painful lesions often accompanied by malodorous discharge. Importantly, HS can have a profound negative effect on patients’ quality of life and put patients at increased risk of anxiety and depression. Although treatments are available, there is a lack of high-quality studies supporting their efficacy, and many patients’ HS is not sufficiently controlled. Health-related online forums, such as Reddit, have become a popular outlet for patients to seek support and recommendations for disease management. Our analysis of patient questions posted to the HS subreddit revealed that most patient questions were related to disease management, with non-pharmacological management being the most asked-about category. Providers can address this potential gap in patient education by inquiring about helpful products and practices from their patients and serving as a conduit for specific recommendations that align with the literature.
